# Guardians of the gut: influence of the enteric nervous system on the intestinal epithelial barrier

**DOI:** 10.3389/fmed.2023.1228938

**Published:** 2023-08-25

**Authors:** Marvin Bubeck, Christoph Becker, Jay V. Patankar

**Affiliations:** ^1^Department of Medicine 1, Universitätsklinikum Erlangen, Erlangen, Germany; ^2^Deutsches Zentrum Immuntherapie (DZI), Erlangen, Germany

**Keywords:** enteric nervous system, enteric glia, gut epithelial barrier, intrinsic primary afferent neurons, inflammatory bowel disease

## Abstract

The intestinal mucosal surface forms one of the largest areas of the body, which is in direct contact with the environment. Co-ordinated sensory functions of immune, epithelial, and neuronal cells ensure the timely detection of noxious queues and potential pathogens and elicit proportional responses to mitigate the threats and maintain homeostasis. Such tuning and maintenance of the epithelial barrier is constantly ongoing during homeostasis and its derangement can become a gateway for systemic consequences. Although efforts in understanding the gatekeeping functions of immune cells have led the way, increasing number of studies point to a crucial role of the enteric nervous system in fine-tuning and maintaining this delicate homeostasis. The identification of immune regulatory functions of enteric neuropeptides and glial-derived factors is still in its infancy, but has already yielded several intriguing insights into their important contribution to the tight control of the mucosal barrier. In this review, we will first introduce the reader to the current understanding of the architecture of the enteric nervous system and the epithelial barrier. Next, we discuss the key discoveries and cellular pathways and mediators that have emerged as links between the enteric nervous, immune, and epithelial systems and how their coordinated actions defend against intestinal infectious and inflammatory diseases. Through this review, the readers will gain a sound understanding of the current neuro-immune-epithelial mechanisms ensuring intestinal barrier integrity and maintenance of intestinal homeostasis.

## 1. Introduction

### 1.1. The enteric nervous system architecture, composition, and epithelial communication

The enteric nervous system (ENS) encompasses two interconnected layers that span the entire length of the intestines: the myenteric (Auerbach’s) plexus and the submucosal (Meissner’s) plexus (1). These layers are anatomically separated by the circular muscle layer, and the myenteric plexus plays a crucial role in coordinating the motor movement of the gut.

#### 1.1.1. Overview of enteric neuron subtypes

Comprised of a network of large unmyelinated neurons and enteric glial cells, the myenteric plexus forms an extensive ganglionic structure. Within this network, excitatory (PEMN/eMN) and inhibitory (PIMN/iMN) motor neurons innervate both the circular and longitudinal muscles and are connected by interneurons (PIN/IN). Sensory neurons (PSN) extend through the circular muscle layer, innervating the submucosal plexus as well as the epithelial layer. With the advent of single cell transcriptomic technologies four major studies have reported the single cell transcriptomes of the human and mouse ENS. Following clustering and annotation of neuronal function, combined with cluster-labelling gene expression patterns, there have emerged seven distinct subtypes. Although studies have found common cell clusters, the readers should note that the annotation of these clusters varies between studies. This highlights the lack of a consensus for an integrated nomenclature and annotation of common clusters emerging from multiple studies.

Additionally, there are intramuscular glial cells situated directly between the muscle fibers. The submucosal plexus, located beneath the circular muscle layer, primarily innervates the submucosal layer and the surrounding crypts of the enteric epithelium. It receives input from primary afferent neurons (IPAN) or secretomotor/vasodilator neurons (PSVN) and exhibits a more compact network with fewer cell bodies compared to the myenteric plexus. The main function of the submucosal plexus is to regulate secretory activity (1). Although the ENS operates autonomously, it maintains innervation connections with the central nervous system through the vagus nerve and prevertebral ganglia (2). It also interacts with stromal cells, interstitial cells of Cajal, and immune cells, including scattered single glial cells throughout the enteric tissue. Thus, there has been a growing interest in understanding the neuronal regulation of the immune system in the context of intestinal inflammatory disorders and for the advancement of cell-based therapies for aganglionic gut motility disorders (3, 4). To facilitate a better understanding of the currently recognized enteric neuronal subtypes, we have provided a comparative Table 1 as a reference guide for neuronal subtypes identified by single-cell transcriptomic studies.

**Table 1:** Molecular taxonomy of the human and mouse ENCs based on single cell transcriptomic studies.

Wright et al. (5)	Drokhlyansky et al. (6)	Zeisel et al. (7)	Morarach et al. (8)	Neuron type	Key genes
Chat 1	PEMN 1, 3, 4, 6	ENT5	ENC1,7	Intrinsic sensory neurons, Interneurons	Chat, Slc18a3, Tac1, Calb2
Chat 1	PEMN 1, 3, 4, 6	ENT5	ENC1,7	Intrinsic sensory neurons, interneurons, mechanosensitive	Chat,Slc18a3, Tac1, Piezo1
Chat 2	PEMN 2, or PIN1, PIN2	(ENT6-7) No equivalent cluster	ENC2-4	Interneurons or excitatory motor neurons 1	Chat, Tac1, Penk
Chat 3 (Met)	PIN1, PIN2 or PEMN 2	(ENT6) No equivalent cluster	ENC4	Interneurons or excitatory motor neurons 2	Chat, Met, Penk, Tac1
Chat 4 (Vglut2)	PIN3 or PSN3	ENT7	ENC12 > ENC7	Interneuron	Slc18a3,Chat, Nos1, Vip, Calb1, Penk, Nefm, Slc17a6
Calcb	PSN1	ENT9	ENC6 > ENC5	Intrinsic sensory neuron	Calcb, Nefm, Scn11a, Calb2, Tacr1, Htr3a, Htr3b, P2rx2, Nmu, Grp, Avil
Nos 1	PIMN1-7	ENT2 > ENT1	ENC8-10	Inhibitory motor neurons 1	Nos1, Vip, Gal, Npy, Htr3a, P2rx2
Nos 2	PIMN1-7	ENT1,3 > ENT2	ENC8-10	Inhibitory motor neurons 2	Nos1, Vip, Gal, Npy
Adult colon	Adult Colon	Adult small intestine	Adult small intestine		

#### 1.1.2. Overview of enteric glial subtypes

Recent single cell transcriptomic studies have identified four (9) to seven (7) major enteric glial cell types, which provide support to the various types of neurons and interneurons, along with enteric mesothelial fibroblasts derived from the neural crest. During prenatal development, these glial cells originate from neuroblasts, with sacral neural crest cells contributing to the development of posterior intestinal enteric neurons and glial cells (10). Signaling molecules such as GDNF and NT-3 are essential for the development of the ENS in an age-dependent manner, with lower efficacy in inducing ENS cell development in older individuals (11). A brief summary of anato-morphological and transcriptomic composition of enteric glial cells can be found in Box 1 and Table 2, which shows the anato-morphological and single-cell transcriptomic classification of enteric glial subtypes.

**Table 2:** Molecular taxonomy of mouse and human enteric glial cells.

Mouse EGC			
Ziesel et al. (7)	Drokhlyansky et al. (6)	Cell annotation	Genes enriched
Small intestine	Colon		
ENTG1	Glia1/2	Proliferating	*Gfra2, Frmd4a, Sox12*
ENTG2	n.a.	n.a.	n.a.
ENTG3	Glia2	n.a	*Tmem200c*
ENTG4	n.a.	n.a.	n.a.
ENTG5	Glia1/2	n.a.	*Slc18a2, Scn7a*
ENTG6	Glia1/2	n.a.	*Lbp, Slc18a2, Scn7a*
ENTG7	Glia2/3	n.a.	*Slc18a2, Fam184b, Lsamp, Otor*
ENMFB	Glia3	Enteric mesothelial fibroblasts	*Ntsr1, Pdpn*

Human EGC			
Elmentaite et al. (10)	Drokhlyansky et al. (6)	Cell annotation	Genes enriched
Developing colon	Colon		
Glia1(DHH+)	Glia2, Glia3, Glia 4, Glia5	n.a.	*DHH, RXRG, NTRK2, MBP, MPZ*
Glia2(ELN+)	Glia1, Glia2, and Glia3	n.a.	*TELN, TFAP2A, SOX8, and BMP8B, MPZ*
Glia3(BCAN+)	Glia1 and Glia6	n.a.	*BCAN, APOE, CALCA, HES5, FRZB*
Differentiating glia	Glia1, Glia2, Glia5	Differentiating glia	*NRXN1*

First two columns refer to specific studies to draw equivalence between clusters from different studies. Readers should note that the study by Elmentaite et al. investigates human developing colon post conception week 6–17.

**BOX 1**: Anato-morphological classification of mouse EGCs: elucidated by Boesmans et al. (12, 13).

- Type I: highly branched, irregular pattern, “astrocyte-like” EGC, in contact with multiple neurons. 70–80% express GFAP, S100B and SOX10 expression is conserved.
- Type II: fibrous interganglionic connections, contact to neural fibers, but no ensheathing. ~50% express GFAP, S100B and SOX10 expression is conserved.
- MP/SMP Type III: sit in extraganglionic regions, up to four major processes with secondary branching running along neuronal processes or small blood vessels. 20–30% express GFAP, S100B and SOX10 expression is conserved
- Type IV: bipolar morphology sitting within the circular and longitudinal smooth muscle layers along nerve fibers. Expression pattern: N/A.
- Mucosal glia: sit within the lamina propria of the mucosa, similar morphology as M/SMP associated Type III. GFAP Expression 20 to 30%, S100B and SOX10 expression is conserved.

Furthermore, we strongly recommend the readers to visit two outstanding and recent reviews to gain an appreciation of the breadth of the developments that have occurred in the ENS field (14–16). These two reviews collectively summarize the discoveries from the initial description of neuronal morphotypes by Dogiel to the recent -omics driven transcriptomic classification of neurons and glial cells of the ENS (14, 15).

### 1.2. The gut epithelial barrier components that maintain host-environment homeostasis

#### 1.2.1. Intestinal epithelial cell composition

**Figure 1:**
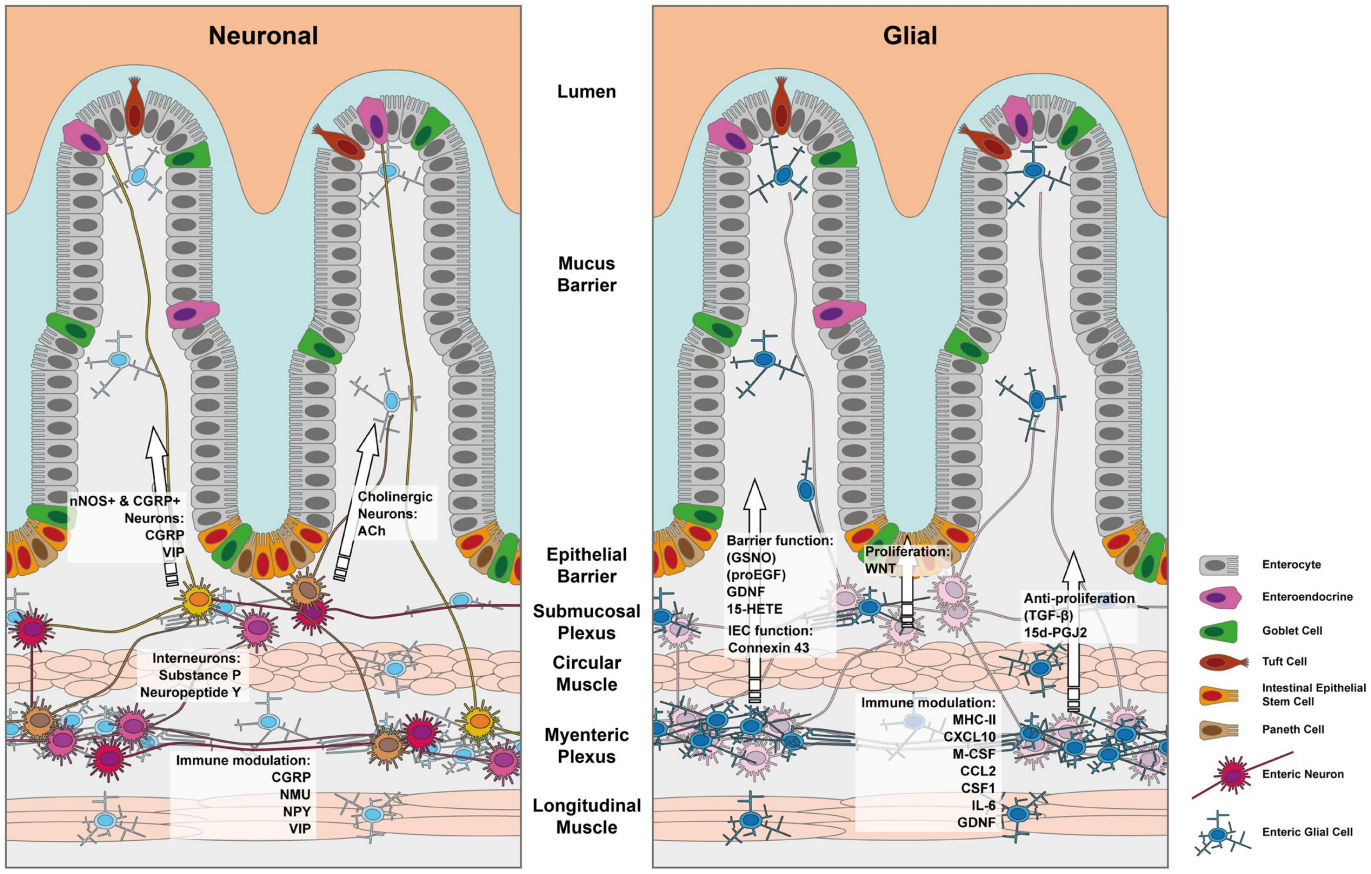
Depiction of the enteric nervous system and its components which influence the IEC barrier. The left panel depicts the effects mediated by enteric neurons and the right panel those exerted by enteric glia. Note that indirect effects via immune cells have been left out for simplicity.

The intestinal epithelial barrier, found in both the large and small intestine, is a remarkably dynamic tissue that undergoes self-renewal every 4–7 days. This continuous renewal process is made possible by a cluster of stem cells located at the base of the intestinal crypts. Within this niche, intestinal epithelial cells (IECs) undergo proliferation and differentiation, giving rise to various specialized cell types as depicted in Figure 1.

The maintenance of the IEC stem cell niche has been shown to heavily rely on WNT signaling from enteric glial cells of the enteric nervous system (ENS), as demonstrated in the study conducted by Baghdadi et al. (17). As IECs proliferate, they gradually move upwards within the crypt-villus structure until they reach the apex, where they reach full maturation. Upon completion of their lifespan, these mature cells undergo controlled cell death, known as anoikis, and are subsequently shed from the intestinal barrier, making room for new cells.

Among the IEC stem cells within the intestinal crypts, Paneth cells play a significant role by producing antimicrobial peptides that protect against potential pathogens and influence the microbiota. Additionally, they contribute to the regulation of stem cells through the secretion of Wnt molecules and metabolic intermediates. Enterocytes, the most abundant cell type among the IECs, specialize in nutrient absorption and the release of enzymes into the lumen. Goblet cells, crucial for the barrier function, produce mucins that serve as a physical barrier against the intestinal microbiome. The thickness of the mucus layer increases from the small to the large intestine in response to higher microbial pressure. Enteroendocrine cells, which engage in local signaling and chemoreception through hormones or peptides, directly interface with the ENS and the innate immune system to aid in microbial defense. They also serve as sources of important neuropeptides, including NPY, PYY, CCK, STT, GIP, GLP-1, GLP-2, among others. Lastly, Tuft cells, although rare, play a critical role in defense and chemosensation, particularly in response to parasitic infections. There is some evidence that Tuft cells may also interact with enteric neurons, however this is a hotly debated topic and several groups are trying to elucidate the existence of an IPAN-Tuft cell axis (18).

#### 1.2.2. Overview of the intestinal epithelial barrier

The intestinal epithelial barrier relies on the formation of tight junctions (TJs) between individual cells. These TJs serve as the primary physical barrier that prevents the invasion of the microbiome into the host. They create a diffusion barrier for metabolites and maintain the polarity of the monolayer by acting as a fence for membrane components. The key proteins involved in TJ formation are claudins and occludin. Occludin, an integral membrane protein, consists of four transmembrane domains. Its N- and C-termini are located on the intracellular side of the membrane, resulting in one intracellular loop and two extracellular loops. These extracellular loops attach to neighboring cells through their respective occludin domains on the extracellular side. Claudins exhibit a similar structure, with one intracellular and two extracellular loops. However, they are more structurally and functionally diverse, with 23 genes identified in humans. In mammals, there are 27 known types of claudins, which exhibit high structural conservation but not genetic conservation. The composition of claudins in TJs varies, contributing to the specificity of ion diffusion. For example, in the blood–brain barrier, claudins create a more specific diffusion barrier, while in the intestinal barrier, they allow for less specific ion diffusion. Claudins are linked to the cellular cytoskeleton scaffold through proteins called zonula occludens (ZO). For a more detailed understanding of the structure and function of TJs and their associated proteins, we recommend referring to the reviews by Zihni et al. (19) and Otani and Furuse (20).

## 2. The enteric glia and the maintenance of the gut barrier

### 2.1. Direct modulation of epithelial barrier function by EGCs

#### 2.1.1. Enteric glial-ablation and impact on gut barrier homeostasis

Research over the past two decades, has uncovered a wealth of information on the important role that enteric glial cells (EGCs) play in maintaining the integrity and function of the intestinal epithelial barrier. In particular, studies have shown that S-100β-immunoreactive EGCs form dense networks around the intestinal epithelial crypts, and that in co-culture settings; EGCs were able to inhibit the proliferation of the transformed IEC cell line Caco2. This effect was found to be due to EGC-derived TGF-β, as the addition of an anti-TGFβ antibody to the co-cultures effectively nullified the anti-proliferative effect exerted by the EGCs on the Caco2 cells (21).

Interestingly, *in vivo* ablation of EGCs via an injection of ganciclovir in transgenic mice expressing the Herpes Simplex Virus type 1 (HSV)-thymidine kinase (tk) gene under the *Gfap* promoter was associated with an increase in the incorporation of tritiated thymidine into epithelial cells (21, 22). Other studies used the same model of EGC ablation and identified specific EGC-derived molecules that are involved in maintaining IEC barrier function, promoting epithelial cell proliferation and differentiation, and protecting IECs from pathogens. The authors showed that EGC-derived S-Nitrosoglutathione improves IEC barrier function by regulating the expression of tight junction-associated protein F-actin. Exogenous administration of S-Nitrosoglutathione rescued the intestinal inflammation and barrier dysfunction in mice where EGCs were ablated and prevented *Shigella flexneri* invasion of IECs (23, 24). In addition, EGC-derived S-Nitrosoglutathione was shown to protect against cytokine-induced barrier defects in an *ex vivo* co-culture model of IECs with EGCs, via the increased expression and localization of occludin and ZO-1 (25). Van Landeghem et al. identified proEGF as another EGC-secreted factor that promotes IEC healing using a combination of techniques involving EGC ablation via the HSV-tk system and *in vitro* assessment of the effects of the EGC-like cell line JUG2 on Caco2 monolayer proliferation in a scratch assay (26).

However, later studies that systematically analyzed the effect of EGC ablation independently of HSV-tk or the *Gfap* promoter identified off-target toxicity of the ganciclovir derivatives on adjacent cells as the main cause of the effects reported in this model, rather than EGC ablation as previously suggested (22, 27, 28). In addition, outstanding work on the classification of EGC subtypes has shown that only a subpopulation of EGCs expresses GFAP (9, 29). This implies that GFAP-driven recombination does not extend to the entire EGC population. Therefore, caution should be exercised in interpreting the conclusions drawn from studies using the HSV-tk-mediated EGC ablation strategy.

#### 2.1.2. Enteric glia and glia-derived factors in the regulation of the epithelial barrier

Furthermore, other studies continued to identify various EGC-derived secreted factors, which affect the epithelial barrier. Some of these factors are graphically outlined in Figure 1. Enteric glial cell-derived GDNF was shown to ameliorate inflammation in a mouse model of barrier damage induced colitis. The authors showed that treatment of mice with recombinant adenoviruses to overexpress GDNF ameliorated dextran sulfate sodium (DSS)-induced colitis, improved colonic transit defects, and *in vitro* IEC healing responses (30–32). The role of GDNF in promoting intestinal barrier integrity was further confirmed *in vitro* using a co-culture model employing EGC and rat IEC cell lines (33). Mechanistically Meir et al. showed that GDNF binding to the RET receptor was important in the stabilization of the desmosomal protein desmoglein 2 in Caco2 cell membranes. Furthermore, the authors reported that inflammatory bowel disease and experimental colitis were associated with a reduction in the expression of GDNF and that restoring GDNF was sufficient to inhibit the inflammation-induced compromise in the epithelial barrier both *in vivo* and *in vitro* (34). In a separate study, Meir et al. used *Gfap*-*Cre* driven reporter system to FACS sort EGCs from the myenteric plexus of mice and showed that these cells indeed produced GDNF and the knockdown of GDNF in an EGC cell line, abrogated the IEC barrier promoting effects on Caco2 cells *in vitro* (35). However, it should be noted that recent single cell transcriptomic data from the human intestinal stromal cells have identified that apart from EGCs, a few other stromal cell types might also be a potential source of GDNF in the gut (36). Other members of the oxylipin family including prostaglandins can also influence the proliferation and differentiation of IECs. Indeed, Bach-Ngohou et al. reported that human submucosal plexus EGCs express lipocalin and EGC cell lines secrete the PPAR-γ ligand 15-deoxy-12, 14-prostaglandin J2 (15d-PGJ2) (37). The authors found that the EGC-derived 15d-PGJ2 exerted inhibitory effects on Caco2 cell proliferation but promoted their differentiation by upregulating the expression of E-cadherin and intestinal alkaline phosphatase (37).

Furthermore, the inhibition of inducible nitric oxide synthase (iNOS) was shown to enhance the IEC barrier protective effects exerted by EGCs in the context of LPS induced barrier disruption arguing that EGC-IEC NO signaling is detrimental under inflammatory contexts (38). Another study investigating the role of iNOS in regulating electrical stimulation evoked chloride and ion secretion the context of trinitrobenzene sulfonic acid− or DSS −induced colitis. The authors found that colitis dependent abrogation of ion secretion was reversed by the inhibition of iNOS, an effect that was mimicked by blocking EGC function with fluoroacetate. In all three colitis models that the authors tested, fluoroacetate mediated inhibition of EGC functions restored the impairment in electrogenic ion transport (39). Nonetheless, it should be noted that fluoroacetate is a non-specific metabolic poison and as such, the possibility of off-target toxicity to other cells cannot be excluded.

It is worth noting that one of the complications of studies that used isolated primary EGC cultures from the myenteric plexus was the question of culture purity. To address this issue, Soret et al. compared and characterized EGC cultures from human, mouse, and rat longitudinal muscle myenteric plexuses and found that approximately 80% of these cells were GFAP, S100β, and SOX10 immunoreactive EGCs (40). Additionally, the authors confirmed the previously described effects of EGCs on promoting IEC barrier and reducing IEC proliferation using the IEC transformed cell line Caco2 (40).

Although several reports involving the assessment of EGCs effects on the IEC barrier using *in vitro* testing of cell lines were indicative of a beneficial effect robust and direct evaluation of the roles of EGCs in maintaining the epithelial barrier were lacking. Important contributions were made over the last 5 years in our understanding of the effects of EGC on the gut barrier using cleaner *Cre* driver lines and a detailed assessment of barrier function *in vivo* during homeostatic and pathological conditions (27, 41, 42). These studies collectively challenged the paradigm that EGC are necessary for maintaining the IEC barrier. For, e.g., using the *Sox10*-*CreER*^T2^ driver line, which drives recombination in all EGCs, Grubišić et al. conditionally and inducibly knocked out connexin 43 in EGCs. Connexin 43 is crucial for EGC activity and upon its tamoxifen induced removal from IECs, caused an impairment in secretomotor functions by regulating electrogenic ion transport, but had no consequences on the IEC barrier (42). In addition, when Rao et al. specifically ablated, EGCs using transgenic diphtheria toxin subunit A (DTA) expression via the pan-EGC inducible driver *Plp1*-*CreER*^T^, the authors failed to see any observable defects in the barrier integrity and IEC proliferation (27). Based on these findings, one may infer that the EGCs are rather innocuous in mediating barrier changes, but in an interesting twist, Grubišić et al. identified a pathogenic role for the Sox10+ EGCs in barrier modulation. The authors showed that the *Sox10*-*CreER*^T2^ mediated knockout of the adenosine 2B receptor ADORA2B, in EGCs protected against DSS-induced colitis and normalized the mRNA expression and distribution of tight junction proteins (41). The view that EGCs do not influence the IEC barrier was also challenged recently in a study, which investigated distinct roles for the *Gfap*^+^ and *Plp1*^+^ EGC subsets in regulating the IEC barrier (17). The authors ablated EGCs using conditional and tamoxifen-inducible transgenic expression of the DTA in either *Plp1-CreER*^T^, or *Gfap-CreER*^T2^ and performed single cell RNA-Seq to identify specific EGC subsets. The authors elegantly demonstrated the critical role of *Gfap*^+^ pericryptal submucosal EGC, but not the Plp1+ EGC subset in regulating the proliferation of the IEC (17). By using the conditional inducible DTA expression approach, the authors also successfully overcame the previously reported off–target toxicity of the HSV-tk and ganciclovir method for EGC ablation. In addition, the authors also identified that the subset of *Gfap*^+^ pericryptal submucosal EGCs are an important source of WNT signals driving the proliferation of the intestinal stem cells (17).

Collective evidence in the field demonstrates an important direct role of EGC subsets in regulating the IEC barrier by controlling proliferation and differentiation of IECs. Further evaluation of other EGC subsets and their influence on IECs will yield greater insights into the role of EGCs in barrier protection.

### 2.2. EGC-mediated immune cell modulation in the regulation of the intestinal barrier

#### 2.2.1. Enteric glia - adaptive immune interactions and influence on the gut barrier

The discovery of EGCs took place over a century ago, but their contribution to regulating intestinal immunity has remained eclipsed due to their classification as cells that provide trophic and protective support to enteric neurons. The immune functions of EGCs were initially explored after the identification of MHC class II molecules expressed by EGCs in inflamed tissues of Crohn’s disease patients. This raised the possibility of an inflammation-induced exogenous antigen presentation capability by EGCs to CD4+ T-cells in the gut (43, 44). Subsequent studies have shown that human GFAP+ EGCs cultured from non-involved margins of small bowel tumor resections, responded to exogenous enteroinvasive *Escherichia coli in vitro*, via upregulating the MHC-II mRNA and protein expression (45). Recently Chow et al. formally tested the hypothesis whether EGCs are capable of presenting exogenously phagocytosed antigens (46). The authors showed that a combination of IFNγ and LPS drove the expression of MHC-II in mouse GFAP+ EGCs at the mRNA and protein level. However, they could not detect any phagocytic activity in EGCs and concluded that the antigens presented by EGCs on the MHC-II are derived from autophagy (46). Furthermore, the authors showed that the EGC MHC-II molecules were involved in modulating T_H_17 and T_reg_ T-cell subsets in the gut, affecting the regulation of the gut barrier and tolerance (46).

The functional significance of IFNγ signaling on enteric glial cells (EGCs) goes beyond just the upregulation of MHC-II. A recent study by Progatzky et al. has revealed an intriguing aspect of this signaling pathway in the context of helminth-induced intestinal inflammation (47). The authors demonstrated that the injury sustained by EGCs in mouse models and human gut inflammation is associated with an EGC-specific IFNγ transcriptional signature. Through single-cell transcriptomic analyses, they identified a subset of mouse and human EGCs, referred to as EGC2 that expresses high levels of GFAP and exhibits a transcriptional enrichment in the IFNγ response pathway. (47). Furthermore, the authors observed that EGCs produce CXCL10, which recruits CD8+ T-cells, as we have summarized in Figure 1. They also demonstrated that the EGC-specific ablation of the Ifngr2 gene leads to a worsening of infection parameters, a reduction in CD8+ T-cell numbers, and elevated histological damage scores. These findings underscore the crucial role of the IFNγ-EGC axis in the initiation of CD8+ T-cell mediated intestinal tissue repair (47).

#### 2.2.2. Enteric glia - innate immune interactions and influence on the gut barrier

In a recent study by Grubišić et al., it was found that inflammation triggers the production of M-CSF from S100β + EGCs in both humans and mice. This subsequently modulates the proinflammatory phenotypic switch in muscularis macrophages (48). The authors discovered that the production of M-CSF from EGCs was dependent on the Connexin-43 protein and required signaling via TNF-alpha converting enzyme. Additionally, EGC Connexin-43 was found to be crucial for visceromotor responses, as a measure of visceral hypersensitivity, during chronicity of colitis (48). Although changes in gut barrier were not directly measured, the accumulation of proinflammatory macrophages can be detrimental to the barrier function in chronic colitis. This suggests that EGCs may function in perpetuating barrier damage. However, a recent study by Stakenborg et al. indicated a barrier-protective role of EGCs. The authors found that EGCs are involved in polarizing muscularis macrophages to an anti-inflammatory state (49). During early inflammation, EGCs recruit monocytes by producing CCL2, and during the resolution phase, EGC-derived CSF1 is required for the polarization of monocytes to two pro-resolving macrophage subsets, namely the Cd206^+^, MhcII^Hi^, and the Timp2^+^, MhcII^Lo^ subsets (49). The apparent contradiction in pro- versus anti-inflammatory effects of EGCs on macrophages reported by these two studies may be due to the differences in the phase and type of inflammation investigated and the choice of promoters used to drive recombination in the EGCs. used by the two studies are different. Grubišić et al. investigated the inflammatory phase of DSS-colitis using the *Sox10*-*CreER*^T2^ line, whereas Stakenborg et al. describe the effects during the resolution phase of muscularis inflammation induced by surgical manipulation and used the *Plp1-CreER*^T2^ line.

An important study by Dora et al. investigating the macrophage populations in the avian and murine myenteric plexuses has indicated the presence of a myenteric plexus barrier modulated by unique macrophage populations (50). Their study revealed that the GFAP+ EGCs possess the ability to secrete extracellular matrix molecules that, along with the continuous layer of glial end feet, create a protective barrier around the healthy myenteric plexus of the gut. This barrier effectively blocks macromolecules larger than 4 kDa from entering the myenteric plexus. The authors further demonstrated that during experimental colitis, this barrier is disrupted in a macrophage-dependent manner (50). The identification of the myenteric plexus barrier has contributed significantly to our understanding of the host organism’s diverse mechanisms to separate self from the environment.

Previously, EGCs have been demonstrated to be a source of specific cytokines that are implicated in barrier repair and IEC proliferation, as opposed to mononuclear immune cells of the plexus (51, 52). Using purified GFAP+ EGC cultures from rat longitudinal-muscle myenteric plexuses, it has been shown that EGCs are a source of bioactive IL-6, and that the production of IL-6 from EGCs is dependent on IL1-β stimulation (51). In addition, a recent report by Schneider et al. showed functional IL1 signaling on EGCs regulated macrophage activation and enteric gliosis in a model of post-operative ileus (53). This indicates that EGCs can modulate the local immune milieu via cytokine signaling under specific inflammatory states. One of the most potent cytokines which promotes gut barrier function is IL-22. An important study by Ibiza et al. revealed the role of EGC-derived GDNF in the regulation of type 3 innate lymphoid cells (ILC3) in the gut. It was found that ILC3 express the high-affinity receptor RET for GDNF and responds to GDNF via the STAT3-mediated production of IL-22 (54). Knocking out the RET receptor in ILC3s or MYD88 in GFAP+ EGCs led to a reduction in IL-22 production and increased gut barrier damage and consequent inflammation. These findings highlight the important role of EGCs in modulating ILC3s and the intestinal barrier through microbial sensing functions (54).

While the mechanisms underlying how EGCs regulate intestinal immunity are still being explored, emerging studies have revealed the intricate regulatory involvement of EGCs in modulating the cell states or functional responses of multiple immune cells, including macrophages, T-cells, and ILCs that are critical in the maintenance of the intestinal barrier.

## 3. The enteric neurons in mucosal barrier function

### 3.1. Direct modulation of epithelial cells by enteric neurons

#### 3.1.1. Epithelial innervation and modulation of fluid flux

Specific enteric neurons known as Dogiel type II multi-axon bearing neurons have long been considered as cells that directly regulate mucosal functions by innervating the mucosa. However, the exact functional significance of this anatomical finding has only recently become clearer. Direct neuron-epithelial innervations have been difficult to prove, but recent research by Bohórquez et al. has revealed the presence of neuropod extensions from the basolateral surfaces of intestinal and colonic enteroendocrine cells that allow them to directly synapse with mucosal efferent and afferent neuronal innervations (55, 56). These findings raise the intriguing possibility of a direct neuroepithelial circuit that allows the enteric nervous system (ENS) to sense and respond to environmental cues.

Previously, it was thought that cholinergic enteric neurons innervating IECs regulated fluid secretion from colonic IECs and granule release from ileal Paneth cells. Acetylcholine (ACh) was also shown to increase paracellular and transcellular permeability and induce chloride ion secretion across the IEC via muscarinic receptors (57, 58). The barrier protective role of ACh became apparent in a mouse model of severe burn-induced distal organ failure. Severe burns can lead to systemic shock, which has been associated with a compromised gut barrier. Constantini et al. measured the gut barrier function in male BALB/c mice exposed to a severe, 7-s steam burn over 30% of the body surface either with or without the administration of α-7 nicotinic acetylcholine receptor agonists (59). The authors reported a significant reduction in the appearance of 4 kDa fluorescently labelled dextran molecules, in the serum of mice that received the cholinergic agonist compared with those that received vehicle. In mice with a healthy gut barrier, the orally administered 4 kDa dextran is incapable of permeating through the gut into the blood stream (59). Recent discoveries have raised an interesting possibility of non-neuronal sources of ACh in the gut. Tuft cells, a specific type of IECs, can produce ACh, forming a non-neuronal cholinergic system in the gut upon sensing luminal components (60–62). However, relative contributions of the neuronal versus non-neuronal cholinergic systems in the regulation of IEC functions in homeostasis and disease remains to be evaluated.

#### 3.1.2. Neuropeptides and cytokines from enteric neurons in epithelial barrier control

Recent research has revealed the direct effects of specific neuronal factors on the regulation of the IEC barrier via diverse mechanisms. For instance, an important function of nociceptive calcitonin gene-related peptide (CGRP) + enteric neurons that sense commensal microbiota was recently discovered (63). The authors showed that mouse and human goblet cells express the high affinity CGRP receptor, RAMP1 enabling a CGRP-mediated regulation of mucous secretion from goblet cells and protection against experimental colitis coupled with microbial sensing by these neurons (63). A subset of the enteric nociceptive CGRP+ sensory neurons also express vasoactive intestinal polypeptide (VIP). VIP has large effects on the intestinal barrier function by regulating multiple barrier functions. For example, VIP stimulates the release of mucous, induces electrolyte and fluid movement across the IECs, induces ZO-1 tight-junction mRNA and protein expression in IECs, and induces the expression of trefoil factor-3 leading to the stabilization of the mucous layer (64–67). Moreover, human nitrergic neurons, which express VIP, inhibit IEC proliferation and improve barrier integrity in *ex vivo* co-culture systems (68).

A subpopulation of enteric interneurons is Substance P (SP) + and innervate the peri-cryptal submucosal plexuses (6). Interestingly, exogenous SP supplementation protects against acute and chronic DSS-induced colitis and barrier breakdown via the upregulation of intestinal epithelial proliferation (69). Another neuropeptide which has strong direct effects on the IECs is neuropeptide Y (NPY). NPY acts on the IECs via the peptide YY receptors subtype 1, which is expressed highly on colonic absorptive enterocytes, goblet cells, and enteroendocrine cells through which NPY negatively regulates ion transport and secretion.

Apart from neuropeptide-mediated regulation of IEC function, recent research has revealed the role of neuronally derived interleukin-18 (IL-18) in regulating the IEC barrier by controlling the expression of antimicrobial peptides in IECs (70). When the authors knocked out IL-18 in epithelial and immune cells, the control of intestinal *S. typhimurium* remained unaffected. However, upon specific ablation of IL-18 from neuronal cells, bacterial killing and goblet cell antimicrobial peptide expression was derailed (70). Interestingly, the IL-18 expressing neurons were VIP+, CHAT+, and nNOS+ and are most likely a population of peripheral sensory neurons (6). However, the conditions that trigger the expression and release of this neuronally-derived barrier protective factor is currently not known.

#### 3.1.3. Enteric neurons as regulators of post-translational mechanisms of barrier protection

Fucosylation, a crucial post-translational modification of membrane glycoproteins and glycolipids, plays a vital role in the production, function, and integrity of intestinal mucous as well as in maintaining microbial homeostasis. Interestingly, a recent study by Lie et al. shed light on the function of VIP+ intestinal neurons in regulating IEC fucosylation, which in turn regulated microbial homeostasis. (71). The authors demonstrated that activation of VIP receptor 1 on IECs by enteric VIP+ neurons had a significant impact on α1,2-fucosylation on IECs by modulating the expression of fuscosyltransferase-2 and various glycoproteins and glycolipids. The absence of extrinsic vagal gut innervation or the chemogenetic perturbation of the ENS VIP+ neurons altered IEC fucosylation and the balance between opportunistic and commensal microbial communities (71). These findings support previous studies highlighting the crucial role of VIP in regulating intestinal barrier homeostasis, as germline VIP deficiency renders mice more susceptible to DSS- and 2,4-dinitrobenzenesulfonic acid-induced colitis, which can be rescued by exogenous VIP administration (72). Thus, it is evident that ENS-derived VIP directly regulates gut barrier function.

Enteric neurons have a profound impact on the intestinal epithelium by regulating mucous production, differentiation, and ion and fluid exchange. Future studies aimed at understanding IEC innervation and barrier integrity will reveal how dense axonal innervations and neuronally-derived factors impact the IEC barrier.

### 3.2. The enteric neurons modulate intestinal innate and adaptive immunity in maintaining the gut barrier

Recent studies have shed light on the complex interplay between various cellular components and neuropeptides involved in regulating barrier integrity. Macrophages in the muscularis have been found to possess unique characteristics and exhibit distinct polarization states regulated by catecholaminergic signaling. However, the impact of this polarization on barrier integrity remains unclear. Additionally, IPANs have emerged as potential sensors of tissue damage caused by microbial dynamics, with the activation of IPANs potentially mediated by intermediate cells such as Tuft IECs or myeloid dendritic cells. Furthermore, sensory neuropeptides like CGRP and NPY, as well as enteric neuropeptide Nmu, have been implicated in regulating immune responses and barrier defense. This section will discuss some of the key findings that link enteric neurons, immune cells, and the gut barrier.

#### 3.2.1. Enteric neuron-macrophage axis in gut barrier protection

Gabanyi et al. in 2016 demonstrated that macrophages in the muscularis exhibit unique characteristics compared to those in the lamina propria and mucosa. These macrophages have a distinct tissue-protective phenotype which is regulated by norepinephrine signaling through the β2 adrenergic receptors in response to infection (73). The authors showed that infection elicited extrinsic sympathetic nerves to signal to these macrophages. However, the impact of this polarization on the IEC barrier integrity was not directly assessed. It would be interesting to note that taken together with the population of macrophages described as the intraplexial macrophages by Dora et al. (50) the role of enteric neuron – macrophage interaction in maintaining the gut barrier as well as the myenteric plexus barrier needs further exploration.

In studies exploring the gut sensory systems during pathogen intrusion, most focus has been on intermediary cells that respond to pathogen- or tissue-derived molecular patterns. However, it is possible that IPANs directly sense tissue damage caused by changes in microbial dynamics through alarmins like IL-25, given that there is some evidence from single cell transcriptomic studies indicating that both the IL-25 receptor chains are expressed by enteric neurons (6). The activation of IPANs in the context of infection may occur through intermediate cells such as Tuft IECs or myeloid dendritic cells via unknown mechanisms.

#### 3.2.2. Enteric neuron-T-cell crosstalk in the regulation of barrier function

Although the governing mechanisms are unclear, functional neuro-immune effects have been identified. For instance, the sensory neuropeptide CGRP negatively regulates the proliferation of mouse naïve splenic T-cells and the production of IL-2 *ex vivo*, but whether similar effects are exerted on activated lamina propria T-cells in the context of inflammation has not been formally tested (74). Interestingly, a CGRP antagonist was found to worsen the severity of DSS-induced colitis, suggesting a barrier-protective role for CGRP (75). Similarly, in the context of Trichinella spirali infection, an IL-4-producing type 2 lymphocyte - CCK-producing enteric neuron axis is associated with enhanced barrier defence, but the exact mechanism of neuron activation during infection remains unknown (76). On the same lines, a subpopulation of CCK producing enteric neurons were recently transcriptomially classified as the mechanosensitive intestinofugal afferent neurons that co-transcribe the *Il4ra* and can induce intestinal smooth muscle contractions leading to worm expulsion and enhanced barrier defence. The exact mechanism of how these neurons is activated in the context of infection remains to be identified.

Specific mucosa innervating secretomotor neurons express the neuropeptide NPY, the transcript levels of which are induced by TNF treatment in primary enteric neuronal cultures (6, 77, 78). Furthermore, the ablation of NPY has been shown to ameliorate tissue injury and barrier disruption, which occurs due to DSS- or *S. typhimurium*- induced colitis (79). Contrary to these findings, in an adoptive T-cell transfer -induced model of colitis, Wheway et al. demonstrated a putative anti-inflammatory function of NPY on T-cells. When the authors transferred Y1 receptor deficient T-cells into leukopenic Rag1 knockout recipients, this caused aggressive colitis (80). Conversely, when the authors challenged Y1 deficient mice with DSS to induce colitis, or with methylated BSA to induce foot pad swelling, the levels inflammation indicators were lower in the knockout mice compared with the controls. Mechanistically, the authors could dissect distinct effects of NPY which strongly represses T-cell activation but promotes antigen presentation (80). Therefore, over all action of NPY the gut seems to promote inflammation and reduce contractility and secretion, all of which are counterproductive for a healthy gut barrier. Further research is necessary to dissect the exact mechanism of NPY-mediated effects on the gut barrier in pathophysiological conditions.

#### 3.2.3. Enteric neurons and the regulation of the gut barrier via the innate lymphoid cells

Regarding helminth control in the gut, neuromedin U (Nmu) has emerged as an important enteric neuropeptide expressed by a subset of IPANs. The receptor for *Nmu*, *Nmur1* is highly expressed on type 2 innate lymphoid cells (ILC2), which play a crucial role in the regulation of type 2 immunity in the gut. Infections by helminth pathogens like *Nippostrongylus brasiliensis* induce Nmu transcription, leading to elevated IL-13 release from ILC2s, increased mucous production from IECs, and smooth muscle contraction for worm expulsion and barrier protection (81, 82). Recent work has shown that CGRP counters the IL-33 and NMU mediated effector functions and proliferation of ILC2s (83, 84). The authors demonstrated that ILC2s are not only sensitive to, but are also capable of producing this sensory neuropeptide and its genetic ablation correlates with improved helminth clearance from the small intestine. Mechanistically, specific ILC2 subsets express CGRP and its CGRP receptors, which are responsible for limiting ILC2 proliferation and inhibiting IL-13 production, a barrier protective cytokine. Interestingly, CGRP uncouples the regulation of the ILC2 IL-5 and IL-13 production such that IL-5 production is enhanced, whereas IL-13 production is inhibited by CGRP (83, 84). This sensory neuropeptide – ILC2 regulatory axis also plays an important role in regulating anti-helminth responses in the lung, which is another barrier tissue (85).

Intestinal VIP+ neurons also regulate the type 3 innate lymphoid cells (ILC3) via the VIPR2 expressed on the ILC3. When the authors knocked out the VIP receptor 2 gene on RORγt+ ILC3, they observed an enhancement in the production of barrier protective cytokines such as IL-22. Moreover, the chemogenetic inhibition of the VIPergic neurons was associated with a heightened protection from pathology caused by the enteropathogenic bacterium *Citrobacter rodentium*. In addition, the barrier disruption, systemic infection, and organismal death observed by chemogenetic activation of the VIPergic intestinal neurons was reversed by exogenous administration of recombinant IL-22 showing that VIP mediated inhibition of ILC3s critically impacts IL-22 mediated barrier protection (86). In line with these findings, Vu et al. reported that the germline deficiency of the VIP gene or pharmacological antagonism of its receptors, protected mice from the adverse pathological and barrier damaging effects of DSS-induced colitis (87).

## 4. ENS and regulation of the gut barrier in IBD

Excessive diarrhea and arrhythmic gut motility are some of the indications for an altered ENS function during IBD. However, not much information is available on whether ENS alterations themselves can impact barrier function in IBD patients. Most of IBD research is being advanced through patient-derived mucosal biopsies, which fail to recapitulate the changes in the neurons and glia in the myenteric plexus embedded deep within the muscularis layers. At a histological level, myenteric plexitis, hyperplasia, and altered neuropeptide code are a common and well-documented finding in Crohn’s disease and ulcerative colitis, especially in ileal manifestations of Crohn’s disease with structuring fibro-stenosis (88–90). A positive evaluation of myenteric plexitis at the margins of the resection site is predictive of an earlier post-surgical recurrence and reflects functional consequences of such plexitis besides the obvious consequences on neuroinflammation and motility (91–96). Elevated levels of EGCs have been reported in patients with IBD, and elevated GFAP+ EGCs were encountered in the payer’s patches of patients with ileal Crohn’s disease (97). Interestingly, the EGC mediators S-nitroglutathione and GDNF worsened paracellular permeability in Crohn’s disease, but not in non-IBD patients (97). However, the specificity of this finding using multiple glia markers such as S100B or PLP1 was not determined. Interestingly, while profiling the polyunsaturated fatty acids that may be secreted by EGCs, Pochard et al. found that human and rat EGCs produced the lipoxin precursor 15-HETE. They then showed that the levels of 15-HETE secreted by EGCs from Crohn’s disease patients were lower than healthy controls EGCs. In addition, 15-HETE administration improved transepithelial permeability by increasing ZO-1 expression in an *in vitro* assay using Caco2 cell monolayers (98). Another interesting correlation is the simultaneous upregulation of TNF and NPY immunoreactivity in inflamed IBD patient samples (79, 99). Nevertheless, it must be noted that not only NPY but also other neuropeptide transcript signatures in bulk tissues may reflect cumulative expression from enteroendocrine cells and ENCs. Therefore, in contexts where there is no evidence of epithelial erosion, such results should be interpreted carefully and should be clearly ascribable via immunohistochemistry to nerve cells. On the same lines, a recent report showed that inflamed tissues from IBD patients have an elevated NPY immunoreactivity and that pharmacological inhibition of the NPY receptors was able to prevent the release of inflammatory cytokines from IBD patient tissue biopsies, as well as protect against experimental colitis and preserve barrier integrity (78, 100). Further studies which investigate the mechanisms of EGC and ENC mediated control of the intestinal barrier in IBD are warranted.

## 5. Conclusion

In conclusion, emerging evidence has highlighted the roles of the enteric glial and neuronal cells in coordinating the immune and epithelial barrier in the gut so as to detect and respond to potential threats while maintaining homeostasis. The maintenance of the gut barrier is crucial, as its disruption can have systemic consequences. The emerging understanding of immune regulatory functions mediated by enteric neuropeptides and glial-derived factors provides intriguing insights into their contributions to the tight control of the mucosal barrier. Figure 1 summarizes the neuronal and glial effectors covered in this review that impact the gut barrier. Continued research in this field holds great promise for developing targeted therapies to manage intestinal infectious and inflammatory diseases.

## Author contributions

JP conceptualized, wrote, and edited the manuscript. MB participated in writing and was responsible for realizing the graphic visualizations. CB edited and proofread the manuscript. All authors contributed to the article and approved the submitted version.

## Funding

This work was funded by the Deutsche Forschungsgemeinschaft (DFG, German Research Foundation) - TRR241 375876048 (A03, B05), SFB1181 (C05), and individual grants with project numbers 418055832 and 510624836. The project was also supported by the Interdisciplinary Centre for Clinical Research (IZKF: A76, A93, and ELAN P120).

## Conflict of interest

The authors declare that the research was conducted in the absence of any commercial or financial relationships that could be construed as a potential conflict of interest.

## Publisher’s note

All claims expressed in this article are solely those of the authors and do not necessarily represent those of their affiliated organizations, or those of the publisher, the editors and the reviewers. Any product that may be evaluated in this article, or claim that may be made by its manufacturer, is not guaranteed or endorsed by the publisher.

## Abbreviations

ENS: enteric nervous system
PEMN/eMN: peripheral excitatory motor neurons or motor neurons
PIMN/iMN: peripheral inhibitory motor neurons or inhibitory motor neurons
PIN/IN: peripheral interneurons or interneurons
PSN: peripheral sensory neurons
EGC: enteric glial cells
IEC: intestinal epithelial cells
ENC: enteric nerve cells; IPANs, intrinsic primary afferent neurons
